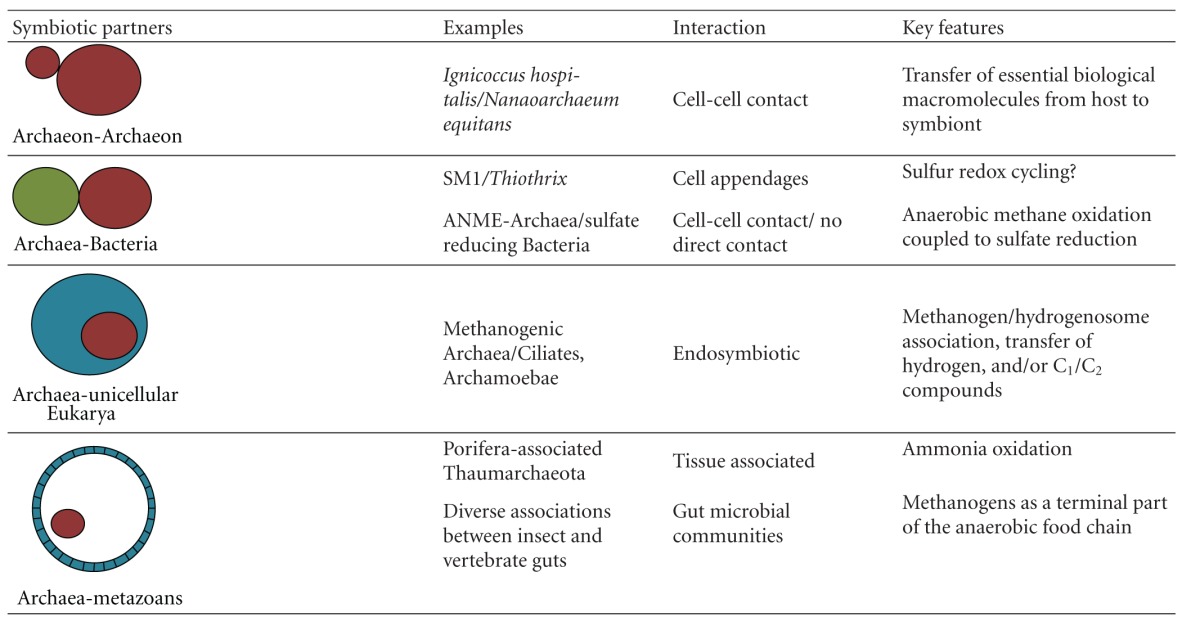# Archaea in Symbioses

**DOI:** 10.1155/2012/596846

**Published:** 2012-12-27

**Authors:** Christoph Wrede, Anne Dreier, Sebastian Kokoschka, Michael Hoppert

**Affiliations:** ^1^Institute of Microbiology and Genetics, Georg-August-Universität Göttingen, Grisebachstraße 8, 37077 Göttingen, Germany; ^2^Hannover Medical School, Institute of Functional and Applied Anatomy, Carl-Neuberg-Straße 1, 30625 Hannover, Germany; ^3^Courant Centre Geobiology, Georg-August-Universität Göttingen, Goldschmidtstraße 3, 37077 Göttingen, Germany

## Abstract

During the last few years, the analysis of microbial diversity in various habitats greatly increased our knowledge on the kingdom Archaea. At the same time, we became aware of the multiple ways in which Archaea may interact with each other and with organisms of other kingdoms. The large group of euryarchaeal methanogens and their methane oxidizing relatives, in particular, take part in essential steps of the global methane cycle. Both of these processes, which are in reverse to each other, are partially conducted in a symbiotic interaction with different partners, either ciliates and xylophagous animals or sulfate reducing bacteria. Other symbiotic interactions are mostly of unknown ecological significance but depend on highly specific mechanisms. This paper will give an overview on interactions between Archaea and other organisms and will point out the ecological relevance of these symbiotic processes, as long as these have been already recognized.

## 1. Introduction

Symbiotic interactions between various groups of prokaryotes as well as between prokaryotes and eukaryotic organisms were one essential driving force of evolution, including the development of differentiated tissues in multicellular organisms [1]. Apart from the essential key events of endosymbiosis, leading to mitochondria and chloroplasts, a multitude of symbiotic interactions at various levels is an ongoing process [2]. Interestingly, most of these interactions are contributed by Bacteria, frequently by Proteobacteria [3]. Regarding parasitic or pathogenic interactions in particular, the outer envelope of the bacterial cell mediates highly specific contact to its host. Surface structures like pili, lipopolysaccharides, and outer membrane proteins may rapidly adapt to modified host tissue structures, mainly with respect to deleterious host-pathogen interactions [4]. How about Archaea? Up to now, no clearly identifiable pathogenic interactions between an Archaeon and its host have been detected, though some archaeal commensals may be indirectly involved in bacterial infections [5]. On the other hand, mutualistic symbioses have been well described, some of them with high relevance to global environmental cycles [6]. Here we will present a short overview on interaction mechanisms known so far and relevant symbioses between Archaea and other organisms. We use the term symbiosis here in a broader sense (see Table 1); in most if not all cases the benefit of both interaction partners could not be proven, though none of these interactions appeared to be detrimental for one of the partners [7].

## 2. Mechanisms for Interaction with Host Cells

Apart from vertically transmitted endosymbionts, any interaction between host and symbiont depends on the surface-surface recognition. A variety of proteinaceous and polysaccharide-based surface structures are known to be involved. Surface layers are common in all Archaean groups known so far. Though their function in adhesion is up to now poorly understood, it may be expected that in particular the glycosylated surface layers are involved in adhesion. In fact, like in Bacteria, extracellular polysaccharides are known as adhesive matrix for biofilm formation [8]. As it has been described for Bacteria, filamentous protein appendages are important for adhesion of Archaean cells. Some of them appear to be unique for particular phylotypes, like the hami, highly complex proteinaceous appendages appearing like hooks. These structures are supposedly involved in the formation of a tight biofilm consisting of the euryarchaeon SM1 and a filamentous *Thiothrix*-related sulfur-oxidizing proteobacterium [9, 10] and seem to be unrelated to any other known surface appendage. Remarkably, the filamentous protein assembly ends up in a terminal hook. In addition, short prickles branch from the main filament. Though it is not known in which way interaction at the molecular level may work, a tight binding of the cells to each other and to various surfaces was shown. The filaments and an exopolysaccharide supposedly excreted by the Archaeon provide the matrix for the formation of a tight consortium between the Archaeon and the sulfur oxidizer. The shape of the consortia may vary but exhibit sort of a “string of pearl” appearance. Each pearl is in the millimeter order of magnitude and is colonized by cells of the anaerobic SM1 Archaeon in the core and a shell of the aerobic sulfur oxidizer. SM1-related sequences have been detected in low saline sulfidic water worldwide, thus a certain ecological significance is likely. The tight association is beneficial for both symbiotic partners when the Archaeon uses the sulfate generated by the sulfur oxidizes for dissimilatory sulfate reduction. The *Thiothrix* “shell” will provide anoxic conditions inside the consortium [11].

In this context another apparently unique surface structure should be mentioned. Though not involved in symbiotic interaction, hollow tubes (cannulae) composed of glycoproteins interconnect the cells of the hyperthermophilic *Pyrodictium occultum* [12]. Another uncommon structure has been described recently by cryoelectron microscopy of a microbial biofilm [13]. Cells of a member of the archaeal group Thermoplasmatales form protuberances penetrating cells of the ultrasmall archaeal Richmond mine acidophilic organism (ARMAN).

In addition to these unique structures, interaction is frequently brought about by appendages that are also common to Bacteria. Several pilus types involved in recognition of and attachment to surfaces have been detected in Bacteria so far [4]. Yet, by far most of the archaeal pili have similarities to just the bacterial type IV pilin. Intriguingly, the archaeal flagellin is homologous to the bacterial pilin protein. The archaeal rotating flagellum is homologous to the bacterial type IV pilus secretion apparatus. Consequently, no homologies between the bacterial flagellar genes and archaeal sequences could be detected up to now [14, 15]. Also the genes of the machinery for pilus assembly have been detected in the archaeal genomes. The involvement of the appendages in attachment may differ in various groups. The flagella of *Pyrococcus furiosus* are probably motility organelles, but are also important for biofilm formation and surface attachment [16]. Flagella and pili are also necessary for the aggregate formation and surface adherence of *Sulfolobus solfataricu*s [17]. As in bacteria, the pili are responsible for primary adhesion on surfaces and initiate biofilm formation. The environmental conditions for biofilm formation have been extensively studied for several *Sulfolobus* strains. Basically, temperature, pH, and iron concentrations, which are also relevant in the natural (hot spring) habitat, strongly influence biofilm development. In particular, pH and iron concentration may synergistically act on biofilm development, but in different ways in various *Sulfolobus* strains [18]. In an artificial archaeal biofilm formed by *Pyrococcus furiosus* and *Methanopyrus kandleri*, the latter adheres to the surface (mica, glass, and others), whereas *Pyrococcus* adheres to *Methanopyrus* via flagella and/or direct contact between cells [19]. *Haloferax volcanii* uses nonmotile pili for surface attachment [20]. 

Pili are also involved in interactions between the Archaea and eukaryote hosts. *Methanobrevibacter*'s polar pilus-like fibers are responsible for the attachment of cells to the hindgut epithelium cuticle of the termite *Reticulitermes flavipes* [21].

Generally, a dual nature of cellular appendages (motility and attachment) is not uncommon and has been also repeatedly described for Bacteria [22, 23]. This feature is also true among Archaea, as the mentioned examples may illustrate. However, some types of cellular appendages have not been detected in Archaea. The type III secretion system (TTSS), in particular, is the essential export mechanism for bacterial flagellins and is also an important pathogenicity factor. Specific bacterial proteins are delivered to a eukaryote host after recognition by the TTSS via a hollow channel. This very specific interaction to eukaryotes may have been developed at a time in evolution, when specific signaling between pathogens and multicellular eukaryotes was evolutionary useful [24].

## 3. Archaeon-Archaeon Interaction

The symbiosis between the host *Ignicoccus hospitalis *and *Nanoarchaeum equitans* is well described at the structural level. *Ignicoccus*, (Desulfurococcales, Crenarchaeota) is an anaerobic, hyperthermophilic obligate chemolithoautotrophic hydrogen oxidizing Archaeon. Interestingly, cells belonging to the genus* Ignicoccus* are surrounded by a dual membrane, which appears to be a similarity to most Bacteria. However, the archaeal “outer membrane” is distinct from the composition of the known bacterial outer membranes. Most interestingly, the outer membrane of *Ignicoccus* hosts the H_2_: sulfur oxidoreductase and ATPase protein complexes, that is, membrane energization takes place at this membrane and not at the inner (normally referred to as cytoplasmic) membrane as it is common in all Bacteria with a double membrane cell envelope [25]. Typical porins, homologous to those in bacterial outer membranes, are missing, which also implies that the *Ignicoccus* outer membrane is not homologous to the outer membrane of Bacteria. Instead, in *Ignicoccus hospitalis*, a unique pore-forming complex (Ihomp1) consists of nine monomers of a small unique alpha-helical protein [26]; other membrane proteins appear to be involved in the *Ignicoccus/Nanoarchaeum* symbiosis as well [27]. The symbiont *Nanoarchaeum equitans* depends obligately on the *Ignicoccus* host. The *Nanoarchaeum* cells are directly attached to the outer membrane of *Ignicoccus*. The extremely reduced genome (490 Kbp) lacks genes for essential biosynthetic pathways, such as lipid, amino acid, and nucleotide biosynthesis. Thus biological macromolecules must be provided by the *Ignicoccus* host; even transfer of ATP from host to symbiont has been discussed [28].

The relationship between *Ignicoccus* and *Nanoarchaeum* does not appear to be a true mutualistic symbiosis: though the growth parameters of either infected or uninfected *Igni-coccus* cultures (containing infected cells of different degrees and uninfected cells) are the same, attached *Nanoarchaeum *cells significantly reduce the ability of *Ignicoccus *to reproduce [29].

Up to now, direct interactions between two archaeal partners appear to be extremely rare. Other species of the genus* Ignicoccus* are free living and could not be infected with the *Nanoarchaeum equitans* symbiont [29]. Though it is unlikely that interactions within the kingdom of Archaea are an exception, it has to be taken into account that interactions between largely unculturable organisms are difficult to detect. In an artificial binary biofilm between *Pyrococcus furiosus *and *Methanopyrus kandleri* hydrogen produced by *Pyrococcus* is utilized by *Methanopyrus*, which implies that mutualistic benefits may lead to stable aggregations between Archaea [18]. Upcoming in situ techniques may uncover interactions between Archaea in the near future [13].

## 4. Archaea-Bacteria Interactions

Under anaerobic conditions, organic compounds are degraded by the anaerobic food chain whereby the product of one group serves as a substrate for the next group within this chain. Methanogenic Archaea terminate the chain by degrading C_1_ and C_2_ substrates to methane and carbon dioxide.

The conversion of higher organic acids to acetate and hydrogen is endergonic, unless the hydrogen partial pressure is kept low. This may be achieved by the activity of hydrogenotrophic methanogens. This necessary coupling of hydrogen formation and uptake by syntrophic microbial consortia is termed “interspecies hydrogen transfer.” A well-known consortium, “*Methanobacillus omelianskii*,” was isolated several times from anaerobic sediments and sewage sludge and was regarded as a pure culture of an anaerobe converting ethanol to acetate and methane [30]. In fact, the culture consisted of a methanogenic archaeon and a Gram-negative Bacterium [31, 32]. Since then a multitude of syntrophic associations have been described, for example, with the fermentative *Acetobacterium* or *Syntrophobacter* [33, 34], with *Desulfovibrio* under low sulfate concentrations [35], but also under thermophilic conditions with *Thermoanaerobacter*, *Desulfotomaculum,* and *Pelotomaculum* [36–38] and with hydrogenotrophic methanogens as syntrophic partners. These examples show the diversity of interactions with respect to organisms and metabolic properties. Though stable aggregates and specific interactions between the syntrophic partners have been observed [39], syntrophy in interspecies hydrogen transfer is generally highly variable and may depend on the availability of substrates [40].

An important process of methane oxidation in anoxic sediments is conducted by consortia of Euryarchaeota and sulfate reducing Bacteria (SRB). The anaerobic oxidation of methane (AOM) has been first postulated by Reeburgh [41]. Up to 90% of methane produced in marine sediments is anaerobically oxidized [42], which makes AOM to an essential process in global methane turnover. However, quantitative modeling based on existing data of the few sampling sites at the ocean floor is still difficult and the contribution of the process to global methane cycling is still a matter of debate [43]. In ocean systems, methane is either generated by methanogenesis in sediments, or abiotically by serpentinization, and may derive from methane hydrates and fossil reservoirs. In cases of high methane fluxes from large reservoirs, the AOM is usually associated with the precipitation of carbonates and sulfides. This has been particularly observed at sites of intense methane seepage, such as marine mud volcanoes and cold methane seeps; also fossil seeps were identified [44–46]. These precipitates are mostly found in sediments as carbonate-cemented plates or large tabular constructions, but also as grapestone-like concretions or even as giant columnar structures, up to several tens of meters in height, buried in the sediment [47–49]. Under anaerobic conditions, below the chemocline of anoxic ocean basins, these precipitates may form tower-like constructions in the water column, reaching several meters in height [50–52]. In most cases, tube-like or columnar towers exhibit cavities that are perfused by methane and seawater. The inner faces of these concretions are covered by remarkably complex biofilms [53–55], dominated by various representatives of the ANME Archaea (ANME: anaerobic methanotroph). The three known ANME groups are not monophyletic. ANME-1 are distantly related to Methanomicrobiales [56], while ANME-2 and ANME-3 are distantly related to Methanosarcinales [57, 58]. A fourth group has been described as ANME-2d or GoM Arc I; this group is not monophyletic with the other ANME-2 subgroups [59–61]. AOM metabolism for this novel group has not yet been proven [42]. ANME-1 and ANME-2 are the most diverse groups detected in a multitude of habitats and appear to be most relevant for AOM in anoxic environments. ANME-1 cells exhibit a cylinder-shaped morphology with an external sheath and were found only in loose association with SRB of the *Desulfococcus/Desulfosarcina *(DSS) group [53]. ANME-2 cells are coccoid and are frequently detected in consortia with SRB [55, 62]. In ANME-2a/SRB-aggregates, both cell types appear to be randomly intermixed, while ANME-2c/SRB aggregates reveal a shell-like structure with SRB at the outer shell of the aggregate. ANME-2 are usually associated with SRB of the DSS group [63, 64], but also associations with alpha-Proteobacteria, beta-Proteobacteria, or *Desulfobulbus*-related SRB and ANME-2 cells without contact to other bacteria were reported [65–71]. 

There is up to now no indication that the metabolism of the SRB in AOM is distinct from free-living sulfate reducing bacteria. The metabolic pathway of the ANME archaea is clearly related to methanogenesis. Intriguingly, ANME Archaea use this pathway in the reverse direction, while reducing equivalents are transferred to SRB [42, 72]. Until now, it seems that AOM with ANME Archaea is feasible just in syntrophy with sulfate reduction. A recently discovered thermophilic ANME group closely affiliated to ANME1 (ANME 1c), though may conduct AOM in contact to hydrothermal vent systems without SRB and with Fe^3+^ as putative electron acceptor. However, conclusive evidence is still missing in this case [73].

The methyl-coenzyme M reductase (MCR) catalyses in methanogenic archaea the terminal step of methane formation. In reversal, MCR is needed in a reversed methanogenic pathway for the initial step of AOM; also most of the other enzyme steps of methanogenesis operate in the reverse direction [74–76]. However, direct evidence for the reverse operation of this pathway is still lacking.

Intermediates for a necessary transfer of reducing equivalents between the syntrophic partners are still unknown. *In vitro *feeding studies excluded hydrogen, formate, acetate, methanol, and even more uncommon compounds like methylsulfides or humic acids [77–81]. The energy yield of AOM is still extremely low, compared with other anaerobic processes [82].

Recent findings indicate that the ANME Archaea are capable of both methane oxidation and sulfate reduction with elemental sulfur as an intermediate [83]. The reduced product HS_2_^−^ may be the disproportionated by the symbiotic sulfate reducers to sulfate and HS^−^. Thus, the symbioses may be less obligate than originally thought.

ANME-2 Archaea in consortia also conduct nitrogen fixation [84]. Nanometer secondary ion mass spectrometry (nano-SIMS) analysis implied the flow of nitrogen compounds from the Archaea to the sulfate reducers. Remarkably, the energy consuming nitrogen fixation is possible even under the conditions of the extremely low energy yield of AOM, though growth rates of the organisms were reduced by a factor of 20. Since AOM is a mayor sink of methane in marine sediments, nitrogen fixation by AOM may be as well a relevant process in the global nitrogen cycle. By this way, carbon, nitrogen, and sulfur cycles are linked by AOM.

Other recently described AOM processes may also be independent of archaeal groups. AOM with iron (Fe^3+^) and mangenese (Mn^3+^, Mn^4+^) has been described for enrichment cultures from marine sediment samples, but a direct involvement of either archaeal or bacterial phylotypes is speculative [85]. A nitrite/nitrate dependent AOM is conducted by Bacteria (NC10 phylum, candidatus *Methylomirabilis oxyfera*; [86]). This process is clearly distinct from ANME/SRB AOM and appears to be homologous to the aerobic methane oxidation of methanotrophic Bacteria.

Another cell-cell interaction between the giant filamentous thaumarchaeote candidatus *Giganthauma karukerense* and a sulfur oxidizing gamma-Proteobacterium has been described recently [87]. A closed cell monolayer of the proteobacteria covers the surface of the large thaumarchaeote filament. It is not known in how far the cells may interact physiologically. It might be possible that the sulfur oxidizer reduces the sulfide concentration in the immediate vicinity of the host cell.

## 5. Interaction between Archaea and Eukarya

With respect to hitherto known mutualistic symbioses with eukaryotes, most but not all Archaea are members of the methanogenic Euryarchaeota. Methanogens are essential in the degradation of organic substrates under anaerobic conditions to methane and carbon dioxide, as terminal part of the anaerobic food chain. It is reasonable to assume that organisms with guts as anaerobic niches of nutrient decomposition harbor also methanogens as commensals. Remarkably, a single methanogen phylotype, *Methanobrevibacter smithii*, is known to be the predominant Archaeon in the human gut microflora [88]. Symbioses between Archaea and eukaryotes, however, are not restricted to the gut anaerobic food chain. Many of the anaerobic protozoa, either free living or gut symbionts themselves, contain methanogenic Archaea as endosymbionts. These free-living protozoa are widespread in sapropels. Instead of mitochondria, they contain hydrogenosome organelles lacking a tricarboxylic acid cycle [89]. Hydrogenosomes are descendants of mitochondria. In these organelles, ATP is generated in a fermentative pathway by conversion of acetyl-CoA to acetate; the reducing power is released as molecular hydrogen [90]. Hydrogenosomes are a prerequisite for the occurrence of endosymbiotic methanogens, and hydrogenotrophic methanogens use hydrogen and carbon dioxide or formate as substrates for methanogenesis [91]. Also acetoclastic methanogens may take benefit from acetate generated by the hydrogenosome [92]. Regularly, these symbionts are transmitted vertically in the protists. Consequently, the phylotypes of the methanogens differ with respect to the protist groups and their habitats. Methanogens from free-living ciliates in freshwater habitats are related to Methanomicrobiales, whereas endosymbionts in ciliates from millipedes, cockroaches, and even frogs are Methanobacteriales [93]. However, during culturing, ciliates may also tend to lose their symbionts and also uptake of *Methanobacterium formicicum* by the ciliate *Trimyema compressum* has been reported [94]. Mechanisms of interactions between the methanogens and protists are largely unknown. Several morphological peculiarities have been described. In the free-living ciliate *Metopus contortus* polymorphic endosymbionts were observed; some cells appear to lose their cell walls and become directly attached to hydrogenosomes [95]. Also variations in size (putatively due to enlargement of cells) and stellate shape of the endosymbiont with close contact to hydrogenosomes have been observed repeatedly [96].

These results show that a peculiar crosstalk between the symbiotic partners is necessary. Like in well-studied symbiosis between eukaryotes and bacterial endosymbionts (including intracellular pathogens), protection against lysosomal digestion or cytoskeletal rearrangements of the vacuole containing the endosymbiont requires elaborate signaling pathways between host and symbiont partners [97]. It is reasonable to assume that the Archaean symbionts possess respective signaling mechanisms. However, the endosymbiotic associations between several groups of protists (Ciliata, and some representatives within groups of Archamoebae) are the only known endosymbioses so far. Thus, this way of interaction with eukaryotes does not appear to be a mainstream in archaeal life styles [98, 99].

Sponges, organisms at the evolutionary basis of the Metazoa, may be described as a diverse prokaryotic community in a eukaryote host, most of the prokaryotes with largely unknown function [100]. Though the majority of the organisms are free living in the sponge mesohyl, endosymbionts are common. Among cyanobacteria and heterotrophic bacteria, also fission yeasts have been described that are maternally transmitted via sponge eggs [101].

Archaea are ubiquitous in marine sponges, sometimes even dominant [102], though their ecological role is poorly understood. Since the composition of the archaeal community is distinct from seawater, a certain specificity of the sponge/Archaeon association must be assumed [103]. Also rather specific associations between certain archaeal phylotypes and sponges have been described. The association between a sponge and the Thaumarchaeota (formerly Crenarchaeota; [104, 105]) *Cenarchaeum symbiosum* has been first described for *Axinella mexicana* [106]. Three species of the Mediterranean *Axinella *harbor filamentous marine “group 1” Archaea colonizing the collagen surrounding the sponge spicules [107]. Marine Euryarchaeota are associated with the demosponge *Tentorium semisuberites* mesohyl [102]. The role of these symbioses is largely unknown, also with respect to the unknown ecological role of the marine Thaumarchaeota. Recent findings imply significance in the sponge nitrogen metabolism [103, 108, 109]. A vertical transmission of the ammonia-oxidizing Archaea also indicates the specificity of the symbiotic relationship [110]. Ammonia oxidizers may utilize ammonia excreted by the sponge as a metabolic end product and may thereby contribute to detoxification of the sponge tissue. This may be in particular of relevance in highly polluted areas, where high concentration of organic compounds and high ammonia concentrations affect marine biocoenoses [103].

The important role of Thaumarchaeota in nitrogen cycling, also with respect to symbioses, has been also identified in some marine mollusks: strains phylogenetically related to *Nitrosopumilus maritimus* were detected inside the tissue of the colonial ascidian *Cystodytes dellechiajei*. Here, nitrification of the Archaeon could be determined in situ [111]. Recent studies on the diversity of ammonia oxygenase genes also show that ammonia oxidizing archaeal communities differ in various coral species and are also distinct from communities in the sediment or in the water column [112, 113]. It must be expected that symbioses between other groups of marine invertebrates and Thaumarchaeota are also of relevance, in particular with respect to ammonia oxidation [114].

Among arthropods, as the largest animal phylum, only in the groups of millipedes, cockroaches, termites, and scarabs relevant methane producing species are present [93]. Methanogens represent the terminal part of the anaerobic food chain in the guts of these insects (especially termites). In this symbiosis, these Archaea utilize the main degradation products hydrogen, carbon dioxide, and acetate released by the previous steps of anaerobic lignocellulose degradation [115]. All methanogens, including the methanogen endosymbiont-bearing ciliates, are in the hindguts of these arthropods [116]. Free-living methanogens adhere to the hindgut wall. Among these big groups, a correlation between a specific diet (e.g., plant litter) and methane production could not be found, and not all members of the mentioned groups contain methanogens. However, in the group of higher termites, soil feeding termites produce more methane and contain more methanogens (according to 16S rRNA analysis) than wood feeders [117]. In the soil feeding species *Cubitermes fungifaber,* the composition of the communities vary across the species, which does not account for a pure vertical transmission of the gut community, but a strong influence of the community in food soil [118]. Remarkably, also a *Natronococcus*-related sequence could be retrieved from the gut. Related strains are obligate haloalkaliphilic organisms of the family Halobacteriaceae, isolated from soda lakes, and are aerobic heterotrophic Archaea [119]. The *Natronococcus-*related strain may be well adapted to the first section of the hindgut (P1 part). This section provides a highly alkaline environment, reaching a pH around 12. For *Cubitermes ortognatus,* an in depth analysis of archaeal communities in four sections of the hindgut revealed remarkable distinctions in particular between the alkaline P1 part and the following P3–P5. Whereas Methanosarcinaceae–related sequences dominated in P1, they were replaced by Methanobacteriaceae-related clones in all other posterior parts of the gut. Interestingly, also Thermoplasmatales and Crenarchaeota contributed up to 40% to the archaeal community in these parts. The ecological role of these archaeal groups have to be elucidated yet.

Methanogenesis in termites is a globally relevant source of methane, with 20–29 Tg methane per year [120]. Methanogens from all ruminants produce 91–107 Tg methane per year, which is the second largest methane source after wetlands. In ruminants, methanogens are in a similar way the terminal part of the anaerobic lignocellulolytic food chains as in termite hindguts and methanogen/ruminant symbioses have been extensively studied. Some methanogens like *Methanobacterium bryantii* or *Methanobrevibacter ruminantium* were isolated from rumen fluids and were extensively studied with respect to biochemistry and energetics in methanogens, including genome analysis of *Methanobrevibacter* [121, 122]. Abundant adhesin-like sequences in the *Methanobrevibacter *genome imply intensive interactions between the methanogen and other rumen microbes. In coculture experiments with* Butyrivibrio proteoclasticus*, several *Methanobrevibacter* adhesins were upregulated and co-aggregates of both cell types were observed [122]. Interestingly, formate utilisation genes were also upregulated. Butyrate, acetate (or lactate), formate, carbon dioxide, and hydrogen gas are major fermentation products of *Butyri-vibrio *during growth on xylan [123, 124]. Hydrogen and formate may be utilized by *Methanobrevibacter* in this syntrophic interaction. In addition to free-living methanogens in the rumen fluid, methanogens which are extra- and intracellularly associated with ciliate protozoa are relevant contributors to methane production. In ruminants, more than one-third of the methane may be produced by these consortia [125].

One might consider that the presence or absence of methanogens in vertebrates generally depends on the diet or the presence of specific anatomical differentiations of the gut and all herbivorous animals harbor the entire anaerobic food chain. Systematic analysis of the methane production in guts of 253 vertebrate species revealed that methane production and hence the presence of relevant amounts of methanogens does depend on the phylogenetic lineage of the animal rather than on the diet or the anatomy of the digestive system [126]. In some phylogenetic lineages like ostriches, intestinal methanogens got lost irrespective of the diet. Methanogens are also missing within the large lineages of Carnivora/Chiroptera/Eulipotyphla (formerly Insectivora), even in herbivorous pandas (Ursidae/Carnivora). Though in all other large lineages methane producers dominate, nonproducers occur also in several “branches” of these lineages. Generally, the results imply that once the methanogens got lost in the course of evolution, they did not reappear in the descending lineages [126]. One special case with respect to the bird digestive system has drawn attention recently. The hoatzin (*Opisthocomus hoazin*) is the only known example for foregut fermentation in birds similar to the ruminants [127, 128]. The rumen methanogens found in hoatzins are more closely related to ruminant strains than to methanogens found in feces of other birds, though the composition of the methanogen community and the phylotypes themselves were still distinct from those found in ruminants [129].

In the intestines of primates, including humans, Archaea are present. *Methanobrevibacter smithii* as the dominant species draws particular attention: a syntrophic interaction between *Methanobrevibacter* and *Bacteroides thetaiotaomicron*, as studied in gnotobiotic mice, may affect the energy balance of the host [130]. *Methanobrevibacter* utilizes the *Bacteroides* fermentation product formate. This syntrophy obviously determines the expression of *Bacteroides* enzymes: the pathway directed towards formate and acetate production is upregulated, whereas alternative pathways towards propionate and butyrate are downregulated. The ongoing human microbiome project will soon update our knowledge on archaeal diversity and putative function in humans.

## 6. Concluding Remarks

Interactions between Archaea and other organisms are definitely as specific as interactions with symbiotic Bacteria prokaryotes. Up to now, the mechanisms of surface recognition are still poorly understood. The prominent “model” pathogens *Escherichia coli, Salmonella typhimurium, Pseudomonas aeruginosa, *and *Vibrio *spp. greatly extended our knowledge on specific interactions of Proteobacteria with animal host tissue. However, model organisms of this kind are still missing in the archaeal world, due to the lack of easy manageable molecular tools for functional studies, in particular with respect to the generation of mutant strains. In addition, we are still far away from even a rough estimate of the true sizes of the large archaeal clades. Hence, we are still unable to explore the diverse ways how Archaea may interact with each other. The description of the few very diverse cases that we know—considering the fundamental differences, for example, between *Nanoarchaeum and Ignicoccus* or the SM1 and sulfur reducer interaction—gives us an impression on the diverse ways how Archaea may interact and how diverse the mechanisms may have to be expected (seeTable 1).

The symbiotic interaction between prokaryotes also leads to the question if the first eukaryote may be an offspring of a symbiotic interaction between an Archaeum and a Bacterium ([131] and references therein). Though the different roles of ancient Archaea and Bacteria are still speculative, it becomes more and more obvious that tight symbiosis between both prokaryotic cell types also direct us to the roots of eukaryote evolution.

## Figures and Tables

**Table 1:** Some examples for symbioses between Archaea and other organisms (red Archaea, green Bacteria, blue-unicellular Eukarya, and metazoans).